# Effect of remimazolam versus propofol anesthesia on postoperative delirium in neurovascular surgery: study protocol for a randomized controlled, non-inferiority trial

**DOI:** 10.1186/s13741-024-00415-6

**Published:** 2024-06-14

**Authors:** Jeayoun Kim, Seungwon Lee, Boram Park, Woo Seog Sim, Hyun Joo Ahn, Mi-Hye Park, Ji Seon Jeong

**Affiliations:** 1 Department of Anesthesiology and Pain Medicine, Samsung Medical Center, Sungkyunkwan University School of Medicine, Seoul, Korea; 2 Biomedical Statistics Center, Research Institute for Future Medicine, Samsung Medical Center, Seoul, Korea

**Keywords:** Neurosurgery, Neurovascular surgery, Postoperative cognitive dysfunction, Postoperative delirium, Propofol, Remimazolam

## Abstract

**Background:**

Remimazolam is a short-acting benzodiazepine newly approved for the induction and maintenance of general anesthesia. Remimazolam emerges as an ideal drug for the neurosurgical population due to its rapid emergence, enabling early neurological assessment, and its ability to maintain perfusion pressure, which is crucial for preventing cerebral ischemia. However, the use of benzodiazepine has been associated with an increased risk of postoperative delirium (POD). There is currently limited evidence about the relationship between remimazolam-based total intravenous anesthesia (TIVA) and POD.

**Methods:**

In this double-blind, randomized, non-inferiority trial, we plan to include 696 adult patients with American Society of Anesthesiologists physical status class I to III, undergoing elective neurovascular surgery under general anesthesia. After informed consent, the patients will be randomized to receive either remimazolam or propofol-based TIVA with a 1:1 ratio. The primary outcome is the incidence of POD within 5 days after surgery. Secondary outcomes include subtypes, number of positive assessments and severity of POD, emergence agitation, intraoperative awareness and undesirable patient movement, intraoperative hypotension, and postoperative cognitive function. The data will be analyzed in modified intention to treat.

**Discussion:**

This trial will evaluate the effect of remimazolam on the development of POD compared to propofol anesthesia. The results of this trial will provide evidence regarding the choice of optimal anesthetics to minimize the risk of POD in neurosurgical patients.

**Trial registration:**

The study protocol was prospectively registered at the Clinical trials (https://clinicaltrials.gov, NCT06115031, principal investigator: Jiseon Jeong; date of first registration: November 2, 2023, before the recruitment of the first participant.

## Background

Neurovascular surgery needs neuronal evoked potential monitoring to provide real-time feedback for possible nerve damage. Waveforms acquired during evoke potential monitoring, especially motor and sensory evoked potential monitoring, can be adversely affected by inhalational anesthetics (Schindler et al. 1998). Therefore, total intravenous anesthesia (TIVA) using propofol was identified as the preferred method of maintenance during neurosurgery which needs neuromonitoring (Walker et al. 2020).

Remimazolam, a new short-acting benzodiazepine, has recently gained approval as an intravenous anesthetic for the induction and maintenance of general anesthesia. It has high water solubility and is hydrolyzed by tissue esterase into an inactive metabolite, which allows a rapid onset of sedation/anesthesia and prompts arousal despite prolonged use. In addition, it could be rapidly reversible by an antidote of benzodiazepine, flumazenil (Kim 2022). In the American Society of Anesthesiologists (ASA) physical status class I and II patients undergoing general anesthesia, the remimazolam-based TIVA has shown comparable efficacy as a general anesthetic to propofol-based TIVA while demonstrating a superior safety profile (Doi et al. 2020). Remimazolam has exhibited a lower incidence of hypotension, reduced vasopressor requirements, and fewer instances of bradycardia compared to propofol-based TIVA (Doi et al. 2020). Remimazolam would be quite appealing in patients undergoing cerebrovascular surgery because early emergence from anesthesia is critically important for early neurological assessment and maintaining perfusion pressure is essential to prevent cerebral ischemia in this population (Teixeira et al. 2024).

Despite these promising properties of remimazolam as a general anesthetic, however, there is currently limited evidence about the relationship between remimazolam and postoperative delirium (POD). It may mitigate POD risk by averting intraoperative hypotension, yet as a benzodiazepine, it could potentially increase the risk (Inouye et al. 2014; Kappen et al. 2022; Vasilevskis et al. 2012; Wachtendorf et al. 2022).

POD is associated with increased morbidity, mortality, and healthcare costs (Inouye et al. 2014). The 1-year survival probability is reduced by approximately 10% for each additional day of POD (Pisani et al. 2009). Additionally, it is closely related to long-lasting postoperative cognitive dysfunction (Goldberg et al. 2020).

Therefore, the investigators designed this prospective, randomized, double-blinded, active comparator-controlled, non-inferiority trial to investigate the incidence of POD after remimazolam-based TIVA compared with propofol-based TIVA in patients undergoing neurovascular surgery. We hypothesized that the use of remimazolam for general anesthesia would be non-inferior to propofol anesthesia in the incidence of POD. In this report, we outline our approach and study protocol and aim to establish an a priori record of our principal methods and primary endpoints.

## Methods

### Ethics and registration

The ethics committee at Samsung Medical Center approved the study (IRB no. SMC 2023–08-135). The study has been registered on ClinicalTrials.gov on November 2, 2023 (NCT06115031). Written informed consent will be obtained from the patients or legal guardians prior to participation in the study. Trained research assistants will inform the patients of study objectives, benefits, and possible risks and how to react if risks occur. They will discuss the trial with patients and obtain written consent from patients willing to participate in the trial. Consent forms are provided for all patients involved in this trial.

### Study setting

**Fig. 1:**
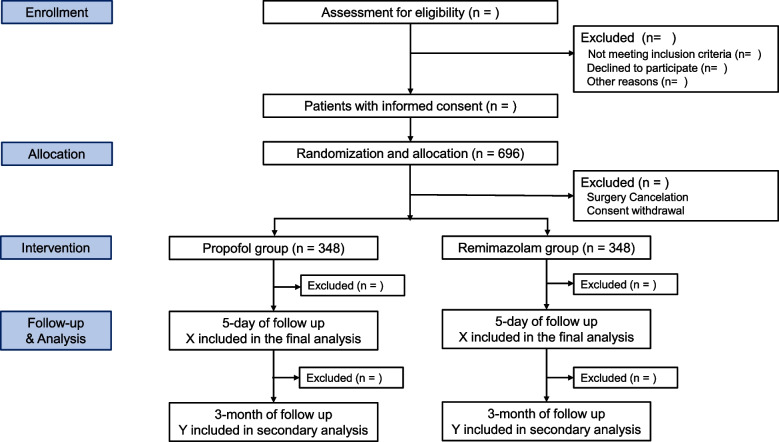
The Consolidated Standards of Reporting Trials flow diagram

This is a single-center, randomized, parallel-group, non-inferiority trial with the patient- and outcome investigator blinding (Fig. 1). The study will be conducted at Samsung Medical Center, tertiary teaching hospital (Seoul City, Republic of Korea, principal investigator: JSJ). The trial will be an investigator-initiated and non-commercial clinical trial. The study design is in accordance with Good Clinical Practice guidelines, the principles of the Declaration of Helsinki, and relevant regulations. The Consolidated Standards of Reporting Trials flow diagram is demonstrated in Fig. 1. The overall schedule is illustrated in Table 1, and the current study protocol is the first version.

**Table 1:** Overall schedule of participants

Timeline	Enrollment	Allocation	Post-allocation							
Timepoint	Within 72 h of admission	Surgery day	*Postoperative day*							
			*0*	*1*	*2*	*3*	*4*	*5*	*7*	*90*
Enrollment:										
Eligibility screen	X									
Informed consent	X									
Allocation		X								
Interventions:										
Propofol group		X								
Remimazolam group		X								
Assessments:										
Baseline variables	X									
Frailty, fall-down, and functionality assessment	X									X
Cognitive assessment (MoCA)	X								X*	X
Intraoperative data		X	X							
Intraoperative adverse events		﻿X	﻿X							
Delirium assessment (CAM-ICU, 3D-CAM)	X		X	X	X	X	X	X		
Quality of recovery				X						
Pain score and opioid requirement			X	X	X	X	X			
Postoperative neurologic and non-neurologic complications			X	X	X	X	X	X	X	X

^*^If the patient is discharged before POD 7, it is performed before discharge

### Patient recruitment

Screening can be performed either in the outpatient unit or in the ward prior to surgery. The lead investigator will be responsible for screening all adult patients who are scheduled for elective neurovascular surgery (aneurysm clipping, microvascular decompression, superficial temporal artery to middle cerebral artery bypass, arteriovenous malformation removal, etc.). A screening log will be compiled to record their eligibility for study participation. Patients who meet the inclusion criteria but do not belong to the exclusion criteria will be contacted by trained research assistants to obtain written informed consent. The study activities were started in November 2023 and are expected to be completed in October 2025.

### Eligibility criteria for participants

Inclusion criteria are (1) patients aged 19 years old or older, (2) ASA physical status classification I to III, (3) elective neurovascular surgery (aneurysm clipping, microvascular decompression, superficial temporal artery to middle cerebral artery bypass, arteriovenous malformation removal, etc.) and receiving general anesthesia.

Exclusion criteria are (1) severe respiratory disease or cardiovascular disease, (2) severe hepatic (Childs-Pugh Score Class C) disease, (3) severe audio-visual impairments that impede communication, (4) hypersensitivity reactions, allergies, or contraindications to the study drugs, (5) evidence of preoperative delirium in Confusion Assessment Method (CAM), and (6) dependency on psychiatric drugs or alcohol.

Dropout criteria are (1) patient withdrawal and (2) surgery cancelation. In participants who withdraw their consent, the reason for their withdrawal will be collected and reported. They will also be asked to specify which aspects of the trial they are withdrawing their consent and participation from. They will be included in the final report of the trial to ensure complete transparency. A participant who withdraws consent for follow-up assessment will be asked about the use of outcome data and only permitted data will be used for the final analysis. All data will be analyzed according to the modified intention to treat (ITT) principle after excluding patients with drop-out criteria.

### Randomization and blinding

Patients will be randomly allocated to either the propofol or remimazolam group in a 1:1 ratio. Block randomization with random permuted block sizes of 4, 6, and 8 was used to ensure a similar number of subjects in each arm over time while minimizing predictability. A randomization code will be generated on the website (sealedenvelope.com) by an independent research assistant who will not be involved in patient care, pack the allocation group in sequentially numbered, opaque envelopes with identical shapes and sizes, and then give the envelope to attending anesthesiologist and nurse. The investigators who will enroll study participants will be blinded to the group allocation.

The group allocation will be blinded to the people as follows: (1) patients: patients are under general anesthesia during the intervention, (2) surgeons: the anesthesia process is initiated prior to the surgeon’s entry into the operating room. This is followed by the placement of opaque drapes that effectively prevent the surgeon from observing any details of the anesthesia technique. The opaque drapes remain in place from the induction of anesthesia throughout the entirety of the surgical procedure, (3) attending anesthesiologists: Anesthesiologists in the operating room cannot be blinded to randomization assignment but are not involved in the study enrollment or assessment of the results, (4) the outcome assessor will be blinded to the group allocation and evaluate the postoperative outcomes. Group allocation is only opened when all the collected data are ready for statistical analysis.

In a medical emergency, such as deterioration of the patient’s condition intraoperatively or adverse events postoperatively, the blindness will be unmasked by the request from the attending anesthesiologist to the principal investigator.

### Preoperative evaluation

Patient’s baseline demographic data will be collected and entered into a predetermined case report form (CRF), which includes the presence of delirium, sensory impairment (Morandi et al. 2021), alcoholism, history of depression, history of falls within 6 months, history of obstructive sleep apnea, level of education, functional status determined by the Barthel Activities of Daily Living index score (Mahoney and Barthel 1965), frailty score (Abellan van Kan et al. 2008), Charlson’s comorbidity index (Quan et al. 2011), type of surgery, neurologic deficit, polypharmacy (Hein et al. 2014), regular use of cholinergic antagonists, dopamine agent, lithium, benzodiazepine drug, floroquinolone, corticosteroid, or opioid analgesics. Preoperative laboratory results, including electrolytes and blood counts, will be recorded.

### Intervention

Patients in the remimazolam group will be administered a loading dose of 0.1 − 0.2 mg/kg intravenously, followed by a continuous infusion of 1 − 2 mg/kg/h until the end of surgery. Flumazenil (100 − 500 mcg) will be administered to wake the patients at the end of surgery until they show eye-opening and simple obeying. Patients in the propofol group will receive general anesthesia using a target-controlled infusion (TCI) of propofol with an effect site concentration of 3 − 5 μg/mL until the end of surgery. In both groups, remifentanil will be co-administered using a TCI mode. Anesthetics in each group will be adjusted by attending anesthesiologists with reference to the bispectral index (BIS), heart rate, and blood pressure.

### Anesthetic care

Patients will not receive any premedication (usually including anticholinergics and sedatives). On arrival at the operating room, the patient will be applied standard monitoring including pulse oximetry, non-invasive arterial blood pressure, and electrocardiography. Anesthesia will be induced and maintained with study drugs with respect to the group allocation, propofol or remimazolam. To facilitate endotracheal intubation, 0.6–1 mg/kg of rocuronium will be administered and mechanical ventilation will be carried out with a tidal volume of 8 mL/kg, inspiratory: expiratory ratio of 1:2, a fraction of inspired oxygen of 0.5 supplemented with air, and positive expiratory pressure of 5 cmH_2_O. The respiratory rate will be modified to maintain normocarbia. The anesthetic depth will be monitored using a BIS monitor (BIS™; Medtronic, Dublin, Ireland) and maintained between 40 and 60. Continuous invasive blood pressure monitoring using an arterial catheter will be performed. Blood pressure and heart rate will be maintained within 20% of baseline during the surgery. Attending anesthesiologists will be discouraged from administering anticholinergics and intravenous hypnotic agents (e.g., midazolam, ketamine, dexmedetomidine, etomidate) except for their study drugs during the maintenance period. In case of bradycardia, glycopyrrolate or atropine will be administered, and their dose will be recorded. At the end of the surgery, 5-hydroxytryptamine 3 receptor antagonists will be administered as prophylaxis for postoperative nausea and vomiting, and 0.01 mg/kg hydromorphone will be administered for pain control. Sugammadex will be administered for the reversal of neuromuscular blockade. After confirming the reversal of neuromuscular blockade and the patient’s obeying, the patients will be extubated and transferred to the ICU. If the patients cannot obey or in case of surgeon’s request, they will be transferred to the ICU with intubated state.

### Postoperative care

Postoperatively, acetaminophen (1 g) or ketorolac (30 mg) will be administered if the patient presents with moderate pain (numeric rating scale, NRS ≥ 4) unless patients have any contraindication for each drug. Additionally, 25 mg of pethidine or 1 mg of hydromorphone will be administered as a rescue opioid. Other perioperative management will be provided according to the institutional protocol.

The researchers will take appropriate measures for injuries deemed to have directly resulted from this clinical trial. In the event of sustained harm, compensation will be offered by utilizing resources in accordance with the participant compensation provisions outlined by the investigator-initiated clinical trial program of Samsung Medical Center. The criteria for compensation disbursement/exclusion will follow the compensation protocol submitted to an institutional review board of Samsung Medical Center.

### Outcomes

#### Primary outcome

The primary outcome is the incidence of POD as defined by any positive assessment between 2 h after surgery and postoperative day 5 or discharge, whichever comes first.

#### Secondary outcomes

Secondary outcomes are (1) severity of delirium, (2) number of positive delirium assessment, (3) emergence agitation which is defined as RASS + 1 or more within 30 min after the endotracheal extubation, (4) delayed extubation (> 1 h after surgery), (5) perioperative adverse events including intraoperative awareness with recall or undesirable patient movement, bradycardia with the use of chronotropic agent, laryngeal spasm, bronchospasm, allergic reaction, arrhythmia, cardiac events (myocardial infarction, vasospasm, or cardiac arrest), or massive bleeding, (6) area under the mean blood pressure threshold (AUT), (7) quality of recovery using Qor-15 questionnaire (Stark et al. 2013), (8) postoperative nausea and vomiting (PONV), (9) incidence and severity of non-delirium complications (non-neurologic or neurologic) within 30 days after surgery, which are graded using Clavien-Dindo classification (Dindo et al. 2004; Landriel Ibañez et al. 2011), (10) length of hospital, defined as the number of days from index neurovascular surgery until initial hospital discharge and ICU stay, defined as the number of hours in the neurosurgical ICU following index neurovascular surgery until the initial ICU discharge, (11) changes in cognition. (12) The occurrence of fall-down and associated injury and functionality at 3 months after surgery.

### Measurement of outcomes

#### Delirium assessments

Trained investigators who are blinded to the group assignment will assess the development of POD using CAM for the Intensive Care Unit (CAM-ICU) and 3-min diagnostic interview for CAM (3D-CAM) for ICU patients and ward patients, respectively. The assessment will be conducted twice a day from the postoperative day 1 until the postoperative day 5 (or discharge whichever comes first) in the morning and the evening, with at least six hours elapsing between assessments. On the day of surgery, delirium assessment will be conducted at least 2 h after the end of surgery. Delirium will be assessed when patients can be aroused sufficiently (Richmond Agitation-Sedation Scale [RASS] >  − 4) (Scale 2003). 3D-CAM is highly sensitive and specific for delirium in hospitalized patients (Marcantonio et al. 2014) and the CAM-ICU is a well-validated method of delirium assessment in intubated patients or in the ICU (Ely et al. 2001). In addition, we adopted structured chart reviews to prevent missing cases during the night shift to detect episodes of delirium (Inouye et al. 2005; Saczynski et al. 2014). This approach increases the sensitivity while maintaining specificity in detecting the incidence of delirium. The severity of POD will be evaluated using the maximum daily score of the Delirium Rating Scale-Revised (DRS-R-98), 0 (no delirium) to 39 (highest severity of delirium) for patients who are screened positive for delirium based on the 3D-CAM or CAM-ICU.

The type of POD will be classified into 3 subtypes (Peterson et al. 2006), which are hyperactive (with a consistently positive RASS, from + 1 to + 4), hypoactive (with a consistently neutral or negative RASS, from 0 to − 3) and mixed type. The severity of delirium will be assessed in the patients who develop delirium within 5 days using the DRS-R-98 and maximum DRS is used in the analysis, 0 (no delirium) to 39 (highest severity of delirium).

#### Cognitive function test

Cognitive assessment will be conducted using Montreal Cognitive Assessment (MoCA) at baseline, 7 days after surgery or at discharge whichever comes first, and 3 months after surgery. Mini-mental state examination is the most widely used cognitive assessment tool for screening and diagnosing dementia; however, it lacks the sensitivity and specificity to detect subtle cognitive impairment (Needham et al. 2017). Therefore, we will use MoCA to detect minimal cognitive impairment (Nasreddine et al. 2005). It comprises six domains: Memory; Executive Functioning; Attention; Language; Visuospatial; and Orientation. Three months later, a telephone interview for the cognitive change will be performed using T-MoCA (Pendlebury et al. 2013), which omits visuospatial, executive, and memory functioning from the MoCA test. The occurrence of fall-down and associated injury and functionality using the Barthel ADL index within 3 months will be assessed using telephone interviews.

#### Adverse events

Perioperative adverse events, which include bradycardia, laryngeal spasm or bronchospasm, allergic reaction, arrhythmia, cardiac events (myocardial infarction, vasospasm, or cardiac arrest), or massive bleeding, will be monitored by the research team and all serious adverse events will be reported to the institutional review board (IRB). The duration and severity of intraoperative hypotension will be assessed using an area under a mean arterial pressure threshold which is defined as the depth below the threshold multiplied by duration, expressed as mmHg * Time (Liem et al. 2020). Awareness with recall will be assessed using the Modified Brice Questionnaire at least 6 h after the end of surgery when the patients are fully alert (Brice et al. 1970). The patient who develops delirium will be assessed following delirium resolution. Undesirable patient movement during surgery will be recorded and graded following criteria. Mild: undesired spontaneous breathing or non-purposeful movement with no impact on the surgery or patient outcome. Moderate: movement that mildly impacted the surgery (e.g., required a pause in the surgery for coughing or straining) and required deepening of anesthesia, deepening of analgesia, or increased muscle relaxant. Severe: movement with a marked negative impact on the surgery (e.g., a patient injury, loss of sterility of the surgical field, purposeful movements suggestive of awareness, or other surgical complication (Wildes et al. 2019).

Postoperative neurologic and non-neurologic complications will be assessed using the example of complication grades proposed to define and grade neurosurgical postoperative complications based on the Clavien-Dindo classification (Landriel Ibañez et al. 2011).

### Perioperative non-endpoint data collection

Patient’s baseline and perioperative data will be collected by investigators who are unaware of the group allocation. Data will be entered into predetermined CRF and a final analysis will be conducted when all the participants finish 3-month follow-up.

#### Preoperative data collection

Sensory impairment (Morandi et al. 2021), alcoholism, history of depression, history of falls within 6 months, history of obstructive sleep apnea, level of education, functional status determined by the Barthel Activities of Daily Living index score (Mahoney and Barthel 1965), frailty score (Abellan van Kan et al. 2008), Charlson’s comorbidity index (Quan et al. 2011), type of surgery, neurologic deficit, polypharmacy (Hein et al. 2014), use of cholinergic antagonists, dopamine agent, lithium, benzodiazepine drug, fluoroquinolone, corticosteroid, or opioid analgesics and preoperative laboratory results, including electrolytes and blood counts will be recorded.

#### Intraoperative data collection

Intraoperative measures, including operative and anesthesia time, anesthetic concentration, perioperative use and dosage of sedatives and opioids, electroencephalographic (EEG) data, hemodynamic data, use of inotropic or vasopressor agents, use of a chronotropic agent for the treatment of bradycardia, total infused volume of crystalloid, transfusion, estimated blood loss, and urine output, will be obtained from patients’ electronic medical records.

#### Postoperative data collection

Pain severity (least and worst) was assessed by NRS from the postoperative day 0 to 5, with 0 (no pain) to 10 (extreme pain). The daily cumulative opioid requirement will be determined from the medical record and quantified using parenteral morphine equivalent (mg) (Ballantyne et al. 2018) until the final delirium assessment is complete. Sleep quality, which is evaluated with the NRS (an 11-point scale where 0 = the worst sleep, and 10 = the best sleep) once daily between 8 and 10 AM) within the 5 days after surgery. The number of valid delirium assessments, along with the reasons why 3D-CAM or CAM-ICU assessments are not conducted, will also be collected.

### Prespecified additional analyses and substudies

#### A. Comparing the incidence and severity of postoperative nausea and vomiting between the remimazolam-based TIVA and propofol-based TIVA

Neurosurgical patients are at high risk for the PONV (ref). Propofol-based TIVA in patients undergoing craniotomies has been associated with a lower incidence of PONV compared to inhalational anesthesia (Chui et al. 2014). Furthermore, a recent meta-analysis demonstrated that perioperative benzodiazepine administration reduced the risk of PONV (Au et al. 2024). In this regard, neurosurgical patients with multiple PONV risk factors are likely to benefit from remimazolam as the primary general anesthetic agent.

#### B. Intraoperative EEG and POD

In the study, EEG data from the BIS monitoring device will be collected in both groups. The intraoperative EEG profile of patients who do and do not develop POD will be compared, and the association with intraoperative EEG and POD will be evaluated.

### Data management

Designated investigators will collect perioperative data and record it into a predetermined CRF. An independent investigator then double-enter the data into a password-protected electronic data set. The protected data set will be backed up every 2 months and each patient will be deidentified using a specific identification number in CRF. After the completion of this trial, the data will be reviewed, all data queries will be completed, and the data set will be locked. An independent statistician will access the deidentified data for statistical analyses according to the predefined statistical plan. The principal investigator is responsible for the accuracy and completeness of data. Internal audits will be done by IRB at Samsung Medical Center. The data will be periodically checked for quality, and an independent data monitoring committee (DMC), which consists of two anesthesiologists who are blinded to the group allocation, will meet twice during the study period and monitor the trial process, data management, and patient safety.

### Safety monitoring

As the administration and dosage of study medications in both anesthesia regimens are within the current clinical practice, we believe that SAEs should be rare. The adverse effects of propofol and remimazolam will be closely monitored during operation by attending anesthesiologists. In case of fetal adverse events, they could stop the infusion of study medications, request the unmaking of group allocation, and inform the principal investigator of any reason for stopping the infusion of study drugs. In addition, a designated investigator will review the occurrence of adverse events possibly caused by study drugs from the start to the end of infusion using chart review. The DMC will perform a review to ensure patient safety. Throughout the study, any adverse events should be reported within 24 h to the DMC.

### Sample size

The primary outcome of this study is the incidence of POD within 5 days after neurovascular surgery. Based on published delirium studies in the neurosurgical population (Harasawa and Mizuno 2014; He et al. 2019; Mokhtari et al. 2020; Morshed et al. 2019; Wang et al. 2020a, 2020b), we estimated the incidence rate and 95% CI as 31.1% (95% CI, 22.9–39.4%). Based on the upper margin of 95% CI, we determined the non-inferiority margin to be 10%. When we plan to enroll an equal number of subjects in each group, a sample size of 330 in each group is needed to detect a difference for a non-inferiority margin of 10%, using a one-sided two-sample *z*-test with unpooled variance, assuming a power of 80%, and a significance level of 2.5%. After considering a drop-out rate of 5%, 348 subjects in each group will be enrolled (a total of 696). The sample size was computed using PASS 2023, version 23.0.1.

Once a patient is enrolled or randomized, the investigators will make every reasonable effort to follow the patients for the entire study period to make the rate of loss-to-follow-up at most 5%. We anticipate high compliance and a low drop-out rate, as all the interventions and sampling in this study will be performed while the patients are under general anesthesia.

### Statistical analysis

All reported study results except adverse events and non-delirium complications are analyzed, following the modified intention-to-treat-principle (ITT). Data will be descriptively presented using summary statistics such as median and quartiles, means and SD, or frequency and percent as appropriate. The standardized mean difference (SMD) will be used for the comparison of baseline data between the randomized groups. Acceptable covariate balance is defined as a standardized mean difference < 0.2 for the entire list of covariates. Repeated measurements, such as NRS score for pain and sleep quality, daily cumulative opioid requirement, and cognitive function will be compared using generalized estimating equation (GEE) analysis among groups.

For the analysis of primary outcome, we will employ a modified ITT principle, which means all randomized participants, regardless of protocol adherence will be included in the primary analysis, except for the case with surgery cancelation and withdrawal of consent after randomization. The incidence among groups will be compared by calculating the 95% CI of the incidence difference: incidence (remimazolam group)-incidence (propofol group), and noninferiority will be achieved if the upper limit of 95% CI is < 10%. The incidence of POD will be compared using a one-sided two-sample *z*-test with an alpha level of 0.025. We will use a multivariable logistic regression model, which will adjust the treatment effect for covariates found to be imbalanced between the two groups.

For the secondary outcome, except for the analysis of intraoperative adverse events and non-delirium complications in which the PP approach will be used, a modified ITT approach will be used. Group comparison will be performed using a two-sample *t* test or Mann–Whitney *U* test as appropriate for continuous variables, and a chi-square test or Fisher’s exact test for categorical variables. We will adjust the effect of imbalanced covariates using a multivariable logistic regression model for count variables and linear regression or quantile regression for continuous variables.

Subgroup analyses will be conducted to assess the consistency of differences in the primary outcome used to differentiate the effect of treatment among different populations (age, sex, surgery type, preoperative cognitive impairment, ASA physical status, duration of anesthesia, alcohol abuse, or preoperative benzodiazepine use within 24 h).

A one-sided *P* value < 0.025 for the primary endpoint and a two-sided *P* value < 0.05 for the secondary endpoints will be considered statistically significant. Missing data will not be replaced, and interim analysis will not be performed. All statistical analyses will be performed by statistical experts utilizing SPSS, SAS, or R software.

## Discussion

In this trial, we aimed to assess the non-inferiority of remimazolam, a new short-acting benzodiazepine, in terms of POD occurrence compared to the conventional intravenous anesthetics, propofol.

Remimazolam demonstrates a favorable pharmacokinetic and pharmacodynamic profile for general anesthesia. However, using benzodiazepines as the principal general anesthetic raises concerns among clinicians about the potential risk of increasing the incidence of POD. Sedation with benzodiazepines in the ICU has been associated with a higher incidence of delirium compared to non-benzodiazepines (dexmedetomidine, propofol) (Casault et al. 2021; Pandharipande et al. 2006; Zaal et al. 2015). Nevertheless, this evidence primarily stems from observational studies. Moreover, long-acting benzodiazepine drugs are more likely to increase the risk of POD compared to short-acting ones. Observational cohort studies have demonstrated no significant increase in the incidence of POD incidence with remimazolam as a general anesthetic in high-risk elderly populations compared to control groups (Aoki et al. 2023; Kaneko et al. 2023). In addition, a recent prospective randomized controlled trial involving 320 patients aged over 60 undergoing elective orthopedic surgery demonstrated a statistically insignificant difference in POD between remimazolam (15.6%) and propofol anesthesia (12.4%) (Yang et al. 2023). Meanwhile, patients in the remimazolam group experienced a lower incidence of post-induction hypotension and required fewer vasoactive drugs intraoperatively. Regarding the risk of POD, evidence from highly qualified randomized controlled trials is necessary for general anesthesia using remimazolam.

Confirming the study hypothesis would provide an opportunity to reappraise benzodiazepine as a principal anesthetic and broaden the options for intravenous anesthetics currently available. It could offer a rationale for adopting a novel drug anticipating several advantages over propofol. Conversely, rejection of the hypothesis would provide insights into potential side effects clinicians should consider when using the new drug.

## Data Availability

Data sharing including the full protocol, individual participant and other relevant study data will be considered upon reasonable request. Only with the permission of the Data Review Board of Samsung Medical Center, the anonymized data will be available from the principal investigator (JSJ).

## Abbreviations

3D-CAM: 3-Minute diagnostic interview for Confusion Assessment Method
ASA: American Society of Anesthesiologist
BIS: Bispectral index
CAM-ICU: Confusion Assessment Method for the Intensive Care Unit
CI: Confidence interval
CRF: Case Report Form
DMC: Data Monitoring Committee
DRS: Delirium rating scale
EEG: Electroencephalogram
ICU: Intensive care unit
IRB: Institutional Review Board
ITT: Intention to treat
MoCA: Montreal Cognitive Assessment
NRS: Numeric rating scale
POD: Postoperative delirium
PONV: Postoperative nausea and vomiting
PP: Per protocol
RASS: Richmond Agitation-Sedation Scale
SAEs: Serious adverse events
SD: Standard deviation
TCI: Target controlled infusion
